# Conscious Sedation in Dentistry for the Management of Pediatric Patients with Autism: A Narrative Review of the Literature

**DOI:** 10.3390/children9040460

**Published:** 2022-03-24

**Authors:** Giulia Vallogini, Paola Festa, Giorgio Matarazzo, Tina Gentile, Annelyse Garret-Bernardin, Gastone Zanette, Angela Galeotti

**Affiliations:** 1 Dentistry Unit, Bambino Gesù Children’s Hospital, IRCCS, 00165 Rome, Italy; paolafesta1@gmail.com (P.F.); giorgio.matarazzo@opbg.net (G.M.); tina.gentile@opbg.net (T.G.); annelyse.garret@opbg.net (A.G.-B.); angela.galeotti@opbg.net (A.G.); 2 Neuroscience Department, University of Padua, 35128 Padua, Italy; gastone.zanette@unipd.it

**Keywords:** autism spectrum disorder, pediatric patient, dentistry, tranquilizing agents, conscious sedation

## Abstract

(1) Background: the variety of autism spectrum disorder makes the definition of guidelines for dental care a challenging task. The aim of this review was to evaluate the literature concerning the use of conscious sedation for dental treatments in pediatric autistic patients. (2) Methods: we searched MEDLINE/PubMed, EMBASE, Cochrane databases in order to identify pertinent studies. The search strategy was based on these areas of interest: autistic spectrum disorder, pediatric patients, dentistry, tranquilizing agents, and conscious sedation. (3) Results: the search yielded 177 non-duplicated articles, of which 24 articles were retrieved for full text review, and 2 were found to address our review aim. The first paper was a retrospective study that included 83 autistic patients sedated either with an oral premedication combined with nitrous oxide/oxygen inhalation or with nitrous oxide/oxygen inhalation alone; the second article was a prospective trial on the effectiveness of 0.3 mg/kg of oral diazepam with 0.5 mg/kg of oral midazolam in 13 sedated uncooperative autistic patients. (4) Conclusions: this review highlights the insufficiency of studies that can provide concrete indications for the dental treatment in conscious sedation of pediatric patients with autism. New studies are needed to better define the appropriate drugs, dosages, sedation level and evaluate patient cooperation.

## 1. Introduction

Autism spectrum disorder (ASD) is a life-long neurodevelopmental disorder with qualitative abnormalities in social interactions and patterns of communication, characterized by a restricted, stereotyped, repetitive repertoire of interests and activities in patients [1].

Patients with ASD show behavioral disturbances like self-injurious behavior, aggression, temper tantrums, psychiatric symptoms, and pica. They are also characterized by mental retardation, seizure disorders, cerebral palsy, fragile X syndrome, tuberous sclerosis, untreated phenylketonuria, neurofibromatosis, and congenital rubella [2].

Patients with ASD may be unable to collaborate during dental procedures. As reported, the main difficulties concern their interaction with dentists and their ability to follow instructions during the visit [3].

The literature has highlighted the complexity of the management of autistic patients and the need to investigate the best ways of providing dental care to these patients. An early diagnosis allowing simple treatments, skills in specific communication with autistic patients, and a long-term follow-up are considered necessary to achieve a greater psychological well-being of the patient and, consequently, a better quality of life [4].

Patients with autism indeed present a particular need for attention because their prevalence of dental caries and periodontal disease is high. A 2016 meta-analysis [5] highlighted precisely this, despite the high heterogeneity of the included articles, showing a difference in caries of 60% and in periodontal disease of 69.4% in the examined pool.

A recent review [6] confirmed that children with ASD have a greater risk of developing caries, periodontal disease, and alteration of the oral microbiota, also adding the element of a greater risk for oral traumas due to their hyperactivity, stereotypes, and self-harm habits. The authors, once again, highlighted the need for personalized approaches with early monitoring as an essential factor to increase patient compliance, identify possible lesions early on, and intervene with an adequate prophylaxis and less invasive treatments.

The use of conscious sedation was evidenced to be a valid support during dental procedures in poorly cooperating children [7].

Whenever patient compliance is an issue and when general anesthesia is contraindicated, also sedative drugs may allow the completion of invasive dental procedures [8].

Usually, general anesthesia is administered for dental treatments to ASD patients, even if the morbidity and mortality risks associated with general anesthesia are considerably higher compared to those linked to conscious sedation [9].

The aim of this review was to evaluate the literature concerning the use of conscious sedation while performing dental treatments in pediatric autistic patients.

## 2. Materials and Methods

### 2.1. Study Design

This paper is a narrative review of the literature considering conscious sedation for dental treatment in patients with autism.

### 2.2. Search Strategy

We followed SANRA (Scale for the Assessment of Narrative Review Articles) guidelines to define the structure of the paper and to evaluate its accuracy [10].

We searched MEDLINE/PubMed, EMBASE, Cochrane databases, in order to detect pertinent studies.

We accomplished the search without restrictions on the language until January 2022.

In PubMed, the following search strategy was used: (((“Autistic Disorder” [Mesh] OR “Autism Spectrum Disorder” [Mesh]) OR “Disabled Persons” [Mesh] OR special needs AND (adolescent [Filter] OR child [Filter] OR preschoolchild [Filter])) AND ((((“Dentistry” [Mesh]) OR “Oral Health”[Mesh]) OR “Mouth” [Mesh]) OR “Dental Health Services” [Mesh] AND (child [Filter] OR adolescent [Filter] OR preschoolchild [Filter]))) AND (((“Benzodiazepines” [Mesh]) OR (“Tranquilizing Agents” [Pharmacological Action] OR “Tranquilizing Agents” [Mesh])) OR “Imidazoles” [Mesh] OR nitrous oxide OR ketamine AND (adolescent [Filter] OR child [Filter] OR preschoolchild [Filter])).

In EMBASE, the following search strategy was used: ((‘autism’/exp OR autism OR ‘disabled person’/exp OR ‘disabled person’ OR ‘special needs’) AND (‘dentistry’/exp OR dentistry OR ‘mouth disease’/exp OR ‘mouth disease’ OR ‘dental procedure’/exp OR ‘dental procedure’) AND (‘tranquilizer’/exp OR tranquilizer OR ‘imidazole derivative’/exp OR ‘imidazole derivative’ OR ‘nitrous oxide’/exp OR ‘nitrous oxide’ OR ketamine OR ‘benzodiazepine derivative’/exp OR ‘benzodiazepine derivative’)) AND ([adolescent]/lim OR [preschool]/lim OR [school]/lim).

In Cochrane, the following search strategy was used: sedation and children, in the topic Dentistry and Oral Health.

As for syndromes related to autism spectrum disorder, we considered every definition that is included in the search tree.

One investigator (GV) compared the final lists of articles and removed duplicates.

The same investigator (GV) screened the titles to remove studies not pertinent to the review; of the relevant papers, the investigator read the abstracts and excluded the studies that were not related to the field of the research.

Therefore, we obtained the full text of all potentially eligible studies, which were fully read in order to exclude those who did not meet the inclusion criteria.

In addition, we carried out a hand search to detect any other possible interesting studies. Any inconsistencies were resolved by consensus with a second investigator (PF).

### 2.3. Criteria to Evaluate the Studies for This Review

We considered all studies designs that involved preschool children, children, and adolescents (up to 18 years old) with a diagnosis of Autism Spectrum Disorder. We only examined studies including human subjects.

As for the interventions, we considered any type of conscious sedation, including pharmacological intravenous (IV) conscious sedation, pharmacological inhalation conscious sedation (INH), pharmacological orally administered (OS) conscious sedation, and intrarectal sedation, as well as a combination of different techniques of conscious sedation associated with dental procedures.

Regarding the outcomes, we included description of dental procedures or successful evaluation of dental treatments in ASD children in papers evaluating preoperative, intraoperative, and/or postoperative time.

### 2.4. Data Collection

Two investigators (GV, GM) extracted key data from the included articles. For each article, we extracted study features, (i.e., study design, year of publication, country), characteristics of the patients (number and age of the enrolled patients), limits and strengths, type of sedation (i.e., nitrous oxide, benzodiazepine), administration route (intravenous, oral, inhalation, intrarectal), type of intervention (i.e., restorations, extractions, combination), preoperative assessment, and measure of anxiety when present.

A third investigator (PF) checked the extracted data.

## 3. Results

### 3.1. Search Results

The search yielded 195 non-duplicated articles. A total of 19 articles were retrieved for full text review, after excluding 148 articles based on the title and 28 after reading the abstracts.

As for the papers excluded on the basis of their title, 23 articles were excluded because they did not include patients with autism, and 125 because they did not regard conscious sedation in dentistry.

As for the papers excluded after reading the abstract, 6 articles were excluded because they did not include patients with autism, and 22 because they did not regard conscious sedation in dentistry.

After reading and extracting data from all the 19 articles, only 2 papers fully addressed our review aim, established at the outset of this scoping review [3,7].

We display the reasons for excluding 17 studies in Table 1.

Some interesting studies [11,12,13,14,15] included autistic patients in the population but considered heterogeneous age groups, and the results were not divided by pathology and age range. Therefore, it was not possible to extrapolate data concerning autistic pediatric patients.

Other studies included disabled patients but did not specify whether they were affected by autism or by other pathologies [16,17,18,19,20].

Four articles were excluded because they did not include patients with autism [8,21,22,23].

One article [24] published a case report of an autistic patient followed over time, but presented the common limits of case reports and was outside the pediatric sphere of our interest for this review.

**Figure 1:**
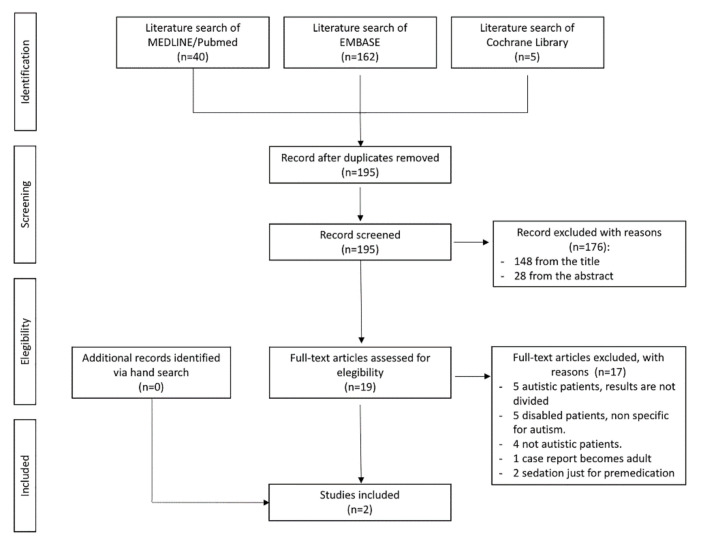
Flow chart.

Two articles were excluded because sedation was used only as a premedication, and the procedures were performed under general anesthesia [1,25] (Figure 1: flow chart).

### 3.2. Study and Patient Characteristics

The analysis included two studies: one retrospective study and one prospective study. We display the characteristics of the included studies in Table 2.

### 3.3. Description of the Included Studies

The first paper [3] was a retrospective study; the first aim was to analyze the dental needs of a group of patients referred to a university dental clinic and the second aim was to investigate key factors influencing the behavioral management. The paper included 100 ASD patients classified at levels 2 and 3 of the Diagnostic and Statistical Manual of Mental Disorders (DSM-5), respectively defined as requiring substantial and very substantial support. The patients included 89 males and 29 females with a mean age of 23.3 years (ranging from 4 to 53 years old), the data were always presented and divided by groups of age: children from 4 to 12 years old, adolescents from 13 to 17 years old, and adults from 18 to 53 years old. Therefore, it was possible to isolate the results related to the group of children and adolescents.

An index to record personal and medical data (general information, medical information, dental care progress, and treatment management) was created for each patient.

At the first visit appointment, almost all children examined presented oral and dental pathologies (90.7%). Particularly, infections of the primary and permanent teeth were found in 24.1% of the subjects, whereas a small percentage presented a malocclusion (7.4%). Children showed more traumas (14.8%) than the older patients.

Regarding dental procedures performed as a treatment plan after clinical evaluation, tooth extractions were more frequent (57.4%); restorations were performed more frequently (31.5%) than supragingival scaling (20.4%) and endodontic treatments (14.8%).

An oral premedication associated with nitrous oxide/oxygen inhalation (46.3%) or nitrous oxide/oxygen inhalation alone (31.5%) was administered; 13.0% of children received the oral premedication as an only sedation measure, and 9.3% of them underwent general anesthesia.

As for adolescent patients at their first visit appointment, almost all of them presented oral and dental pathologies (93.1%). Particularly, 24.1% of these patients presented malocclusions, 13.8% had infections of the permanent teeth, and 10.3 reported bruxism.

Regarding the dental procedures performed as a treatment plan after clinical evaluation, tooth extractions were the most frequent treatment (34.5%) also in adolescents; restorations were also often performed (27.6%), and only a small percentage of the patients underwent supragingival scaling (10.3%), endodontic treatments (3.4%), and subgingival root planning (3.4%).

In the adolescents group, the most frequent approaches were performed after administering an oral premedication associated with nitrous oxide/oxygen inhalation (41.4%) and general anesthesia (48.3%); for only a small percentage of the patients, oral premedication was used alone (10.3%), and none of them was administered only nitrous oxide/oxygen inhalation.

Considering the pediatric patients, dental treatment succeeded with oral premedication only, nitrous oxide/oxygen inhalation, and oral premedication + nitrous oxide/oxygen inhalation in 90.8% of children and 51.7% of adolescents. The other patients were treated under general anesthesia.

The paper did not specify the premedication prescribed together with nitrous oxide/oxygen inhalation and the doses utilized.

The second paper [7] was a prospective, randomized, double-blind, cross-over trial; the aim was to compare the effectiveness of 0.3 mg/kg of oral diazepam with 0.5 mg/kg of oral midazolam in sedating uncooperative autistic patients for dental treatments. The drugs were administered as premedication prior to nitrous oxide/oxygen inhalation.

The sample consisted of a group of 13 autistic patients who demonstrated combative or uncooperative behaviors during the first examination visit and included 10 males and 3 females ranging from 5.8 to 14.7 years of age (mean age 8.68). They underwent two sedation appointments.

The patients were randomly allocated to receive diazepam or midazolam on their first appointment; the alternate drug was administered on the second appointment. Either oral diazepam (0.3 mg/kg, with a maximum dose of 10 mg) or midazolam (0.5 mg/kg, with a maximum dose of 20 mg) was given. The drugs were administered between 45 to 60 min and 20 to 30 min, respectively, prior to treatment.

The patients were treated with nitrous oxide/oxygen inhalation sedation using lidocaine with epinephrine as local anesthesia.

The dental procedures included restorations, endodontic treatments, stainless steel crowns, scaling, primary teeth extractions, sealants and fluoride applications.

The authors defined a total score (sum of three behavior scores within 60 min) that categorized autistic patients in four groups: “very good”, “good”, “fair”, “poor” in function of their behavior in relation to sedation and treatment. The evaluations of “good” and “very good” were classified as “success”, and this result had to be achieved in at least 70% of the patients in order to define the drug effectiveness. In this work, 23% of the patients who received diazepam were classified as “good”, and 54% as “very good”, whereas 23% of the patients who received midazolam were classified as “good”, and 77% as “very good”. These results were considered a success. The patients who received diazepam were rated also poor (8%) and fair (15%), requiring treatment interruption or not rendered and intermittent treatment interruption, respectively.

The study demonstrated that both diazepam and midazolam accomplished safe and effective conscious sedation in autistic patients. Midazolam was more effective at times of increased stimulation (such as injections) in regard to sleeping behavior; similarly, in regard to crying, body movement, and overall behavior, midazolam was more successful, especially in the early phases of treatment. In conclusion, midazolam showed statistically more significant results and appeared to be more effective than diazepam because it showed a higher effectiveness in regulating sleep, body movement, and crying behavior and induced a homogeneous response in the patients, in spite of its shorter effect. On the other hand, diazepam provided a longer duration but a higher variation in behavioral responses.

## 4. Discussion

The retrieved results only allowed a narrative review, since only few articles satisfied the research criteria.

Our literature review evidenced that the majority of the studies did not present complete data according to our research criteria: groups of patients with a confirmed diagnosis of autism, conscious sedation performed for dental treatment, and pediatric patients. The reasons for the exclusion of some articles from our review were detailed in the Results.

In general, we considered only a small number of articles because of the lack of studies in the literature that examined only pediatric patients with a certain diagnosis of autism undergoing dental treatments. In some cases, pediatric patients were examined within heterogeneous groups, but the articles didn’t provided a subdivision of the results that allowed us to extrapolate the data concerning pediatric autistic patients.

Furthermore, conscious sedation was often used only as a premedication before general anesthesia, whereas our aim was to evaluate conscious sedation performed for dental treatments to have indications for the daily clinical management of autistic pediatric patients.

We believe it is important to learn more about conscious sedation for dental treatments in autistic patients, even with respect to the complications appearing after treatments performed under general anesthesia; in a study published in 2021 [26], Train et al. showed, 8 h post-surgery, a higher incidence of adverse behavioral effects, especially difficulties in walking and nausea, in ASD patients with respect to healthy patients.

The need to investigate the topic of conscious sedation for autistic patients also arises from the observation of the difficulties that are encountered during the dental treatment of these patients. As analyzed by Logrieco et al., in 2020 [27], care experiences, from the perspective of both families and professionals, are key to improving clinical practices. In that study, caregivers and dentists were asked to fill in a questionnaire about the difficulties encountered during visit and treatment by children with ASD.

As emerged from the study, children with ASD present difficulties with the dental health care process, resulting in a lower probability of effective treatment. The dentists have to deal with a disorder, such as autism, they only marginally know and that requires a deep knowledge in order to provide these children with the best possible treatment.

It is therefore important that these patients enter a program and that they are evaluated several times. As a matter of fact, it has been highlighted [28] that those children who learn to collaborate in an oral examination will successfully maintain the same behavior in future visits. In addition, they can also acquire skills in some more demanding procedures, which is an important data if we consider the difficulties observed with the general anesthesia.

Therefore, only two studies were included in our review [3,7].

The first article [3] was very relevant because it included only patients with a confirmed diagnosis of autism, divided the patients into age groups so that the results for each category could be analyzed separately, and finally specified in detail the types of treatment and the sedation approach. The limits of the paper were the lack of information about the specific drugs used for oral premedication and their dosage; the lack of information regarding a possible perioperative assessment; the lack of measures of anxiety and behavior. Finally, no data about the type of local anesthesia were reported.

This study [3] highlights that oral premedication and/or nitrous oxide/oxygen inhalation at a high dose represent a good way to carry out conservative dental cares, with a significant efficiency in children. Nevertheless, in a considerable number of cases, especially in adolescents, general anesthesia could not be avoided, though this last approach significantly limited conservative treatments and led to tooth extractions.

The second article [7] was very relevant to our goal because it only included patients with a confirmed diagnosis of autism, and the age range was specific for pediatric patients. It described a detailed clinical protocol of conscious sedation, specifying the dose and timing of drug administration. It also used a behavioral assessment that allowed a detailed analysis of the effects of the drugs used; this attention to the patient’s behavior is very interesting and is found in the literature [29,30], where the importance of this type of evaluation is emerging, also in reference to the help it can provide in the preparatory phase for treatment.

Our research has led to poor results; therefore, we can only report some observations based on what is present in the literature.

Based on our results, the inhalation of nitrous oxide/oxygen may be used to perform dental treatments under conscious sedation in children with ASD, even if it is usually reported as a premedication for general anesthesia. For adolescents, an oral premedication associated with nitrous oxide/oxygen inhalation and general anesthesia might be the two preferable techniques for dental treatments.

As for the premedication, midazolam might preferable to diazepam because it induces a homogeneous response in the patient, in spite of its shorter duration.

**Figure 2:**
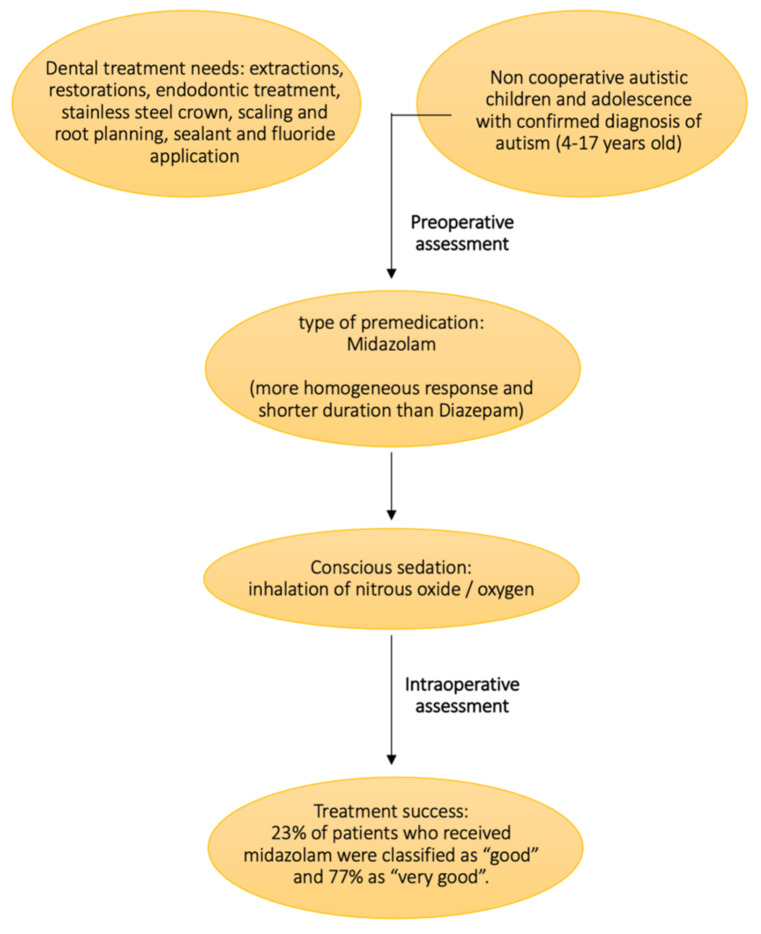
Representation of the findings.

These findings are presented in Figure 2.

Our literature review evidenced that few studies provided clinical indications to manage dental treatments under conscious sedation in pediatric patients with autism.

The limitation our study is that only two relevant articles have been published on this topic, and the conclusions we can draw are limited.

In fact, our goal was to focus on pediatric patients diagnosed with ASD who underwent dental therapies with conscious sedation.

Moreover, some papers did not specify the drugs and dosages used as oral premedication, which is fundamental to define some guidelines for clinicians, and did not consider measures of anxiety and behavior, which are important to understand the specific starting conditions of each patient.

Therefore, further studies are needed to define the types of drug, their dosages, and the most appropriate techniques for conscious sedation for dental care in autistic children. Furthermore, future studies should investigate the efficacy of conscious sedation in dental treatments as a function of the severity of the disease, from a mixed developmental disorder to different degrees of autism.

### 4.1. Bullet Points

- The inhalation of nitrous oxide/oxygen may be used to perform dental treatments under conscious sedation in children with ASD
- Midazolam might be preferable to diazepam in children with ASD
- Future studies about tranquilizing agents should also investigate the efficacy of conscious sedation in dental treatments as a function of the severity of the disease

### 4.2. Future Goals

The issue of conscious sedation must be addressed together with behavioral techniques to increase the collaboration of autistic patients, especially of those who are treated with conscious sedation. The topic is addressed in the literature, but future studies should aim to provide real uniform guidelines for behavioral training techniques to be used in preparation for sedation, in order to increase the success rate and make the approach to dental care less stressful for these patients.

## Figures and Tables

**Table 1:** Reasons for excluding 17 studies [1,8,11,12,13,14,15,16,17,18,19,20,21,22,23,24,25].

Authors	Title	Reason
Braff M.H. et al., 1979	Sedation of the autistic patient for dental procedures	Although if the study involves autistic patients, heterogeneous age groups are included and the results are not divided: therefore, it is not possible to analyse only the results of paediatric patients.
Capp P.L. et al., 2010	Special care dentistry: Midazolam conscious sedation for patients with neurological diseases	Although if the study involves autistic patients, heterogeneous age groups are included and the results are not divided: therefore, it is not possible to analyse only the results of paediatric patients.
Fukuta O. et al., 1993	The sedative effect of intranasal midazolam administration in the dental treatment of patients with mental disabilities. Part 1. The effect of a 0.2 mg/kg dose.	The study includes patients with pathologies including autism and developmental delay of various types, the results are not divided by pathology so it is not possible to isolate and analyse the results for autistic patients.
Faulks D. et al., 2007	Sedation with 50% nitrous oxide/oxygen for outpatient dental treatment in individuals with intellectual disability.	The article deals with dental care in sedation in non-cooperating patients including a percentage of patients with Autistic behaviour or psychiatric disorder (27.8% of the total examined), it is therefore a mixed group, in any case the results are not divided by groups, moreover it does not specify the type of dental intervention.
Yilmaz M.Z. et al., 2014	Anaesthetic management of dental procedures in patients with special needs: A retrospective analysis of 519 patients in North of Turkey	The article deals with patients not only autistic but with various types of delay, the results are not divided by pathology so it is not possible to isolate and analyse the results of autistic patients
Macdonald A.G. et al., 1970	The use of diazepam for conservative dentistry in handicapped children	The text is a summary of the works presented at a conference in 1969, it reports the success rates in the conservative dental treatment on handicapped patients (not specific for which pathology therefore not specific for autism) with intravenous midazolam, droperidol, methohexitone.
Allen W.A. 1984	Nitrous oxide dosage in relative analgesia.	The study includes patients under the generic name of "handicapped patients" so there is no diagnosis of autism, moreover patients from 3 to 59 years old are included and the tables are made up of subgroups of which the one with the younger age group still reaches 20 years, therefore beyond the range considered in the review.
Roberts R.E. et al., 1978	Dental care for handicapped children re-examined: II--dimensions of dental practice.	The study includes disabled patients but does not specify whether autism or other pathologies.
Galeotti A. et al., 2016	Inhalation Conscious Sedation with Nitrous Oxide and Oxygen as Alternative to General Anesthesia in Precooperative, Fearful, and Disabled Pediatric Dental Patients: A Large Survey on 688 Working Sessions	The study includes disabled patients but does not specify whether autism or other pathologies.
Lin I.H. et al., 2021	A comparative study of propofol alone and propofol combined with midazolam for dental treatments in special needs patients.	The study includes disabled patients but does not specify whether autism or other pathologies.
Azevedo I.D. et al., 2013	Efficacy and safety of midazolam for sedation in paediatric dentistry: a controlled clinical trial.	This article includes healthy but uncooperative patients, not autistic patients, it refers to patients who are not cooperating due to age and fear but not with pathologies.
Eid H.J. 2002	Conscious sedation in the 21st century.	This article deals with odontoiatric clinical visits made in sedation, not with dental treatments; the groups of patients in their variability are not analysed, so it is not specific for autism.
Shapira J. et al., 1992	Evaluation of the effect of nitrous oxide and hydroxyzine in controlling the behavior of the pediatric dental patient.	The study does not include autistic patients.
Folayan M.O. et al., 2002	Seminars on controversial issues. A review of the pharmacological approach to the management of dental anxiety in children.	This article reviews the methods of sedation, there is no clinical study, it does not include subjects with autism.
Prakash S. et al., 2016	Premedication in an autistic, combative child: Challenges and nuances	It’s a case report but it cannot be included because sedation is used only as an oral premedication before surgery under general anesthesia.
Stuker E.W. et al., 2018	Third time’s a charm: Oral midazolam vs intranasal dexmedetomidine for preoperative anxiolysis in an autistic pediatric patient.	It’s a case report but it cannot be included sedation is used only as an oral premedication before surgery under general anesthesia.
Baldinelli L., Baldinelli L.G. 1991	An autism patient treated with different anesthesia and sedation techniques over 13 years	Case report, cannot be included because it’s a comparison of the same patient over time, non only in childhood

**Table 2:** Characteristics of the included studies.

	Study, Year	Country	Study Design	Enrolled Participants	Participant Age, Years	Types of Sedations, Route of Administration	Perioperative Assessment of Anxiety	Measure of Anxiety	Measure of Behaviour	Type of Treatment	Treatment Success	Local Anaesthesia
1	Mangione F., 2019	France	descriptive study	83	54 children (4 to 12 years old) 29 adolescents (13 to 17 years old)	10 oral premedication, 17 nitrous oxide/oxygen inhalation, 37 oral premedication + nitrous oxide/oxygen inhalation				restorations, endodontic treatment, teeth extractions, sealant, supragingival scaling,	Dental treatment succeeded with oral premedication only, nitrous oxide/oxygen inhalation and oral premedication + nitrous oxide/oxygen inhalation in 90.8 % of children and 51.7 % of adolescents. The other patients had to be treated under general anaesthesia.	
2	Pisalchayong T., 2005	Thailand	prospective, double blinded, randomized study	13	5–15 years	Diazepam OS 0.3 mg/kg (OS) Midazolam OS 0.5 mg/kg (OS) Then: Nitrous Oxide/Oxygen 50%.(nasal)	Preop, intraop		Overall behaviour score considering sleeping behaviour, body movement pattern, crying behaviour	restorations, endodontic treatment, stainless steel crowns, scaling, primary teeth extractions, sealant and fluoride applications	100% of treatment performed when using Midazolam: patients display Good (23%) and very good (77%) overall behavioural rate. Using Diazepam patients were frated also poor (8%) and fair (15%) with treatment interrupted or not rendered and treatment interrupted intermittently, respectively.	Lidocaine 2% with epinephrine 1:10,000

## Data Availability

Not applicable.

## References

[1] Prakash S., Pai V.K., Dhar M., Kumar A.A. (2016). Premedication in an autistic, combative child: Challenges and nuances. Saudi J. Anaesth..

[2] Loo C.Y., Graham R.M., Hughes C.V. (2009). Behaviour guidance in dental treatment of patients with autism spectrum disorder. Int. J. Paediatr. Dent..

[3] Mangione F., Bdeoui F., Costa M.D., Dursun E. (2020). Autistic patients: A retrospective study on their dental need and the behavioural approach. Clin. Oral Investig..

[4] Herrera-Moncada M., Campos-Lara P., Hernández-Cabanillas J.C., Bermeo-Escalona J.R., Pozos-Guillén A., Pozos-Guillén F., Garrocho-Rangel J.A. (2019). Autism and Paediatric Dentistry: A Scoping Review. Oral Health Prev. Dent..

[5] Da Silva S.N., Gimenez T., Souza R.C., Mello-Moura A.C.V., Raggio D.P., Morimoto S., Lara J.S., Soares G.C., Tedesco T.K. (2017). Oral health status of children and young adults with autism spectrum disorders: Systematic review and meta-analysis. Int. J. Paediatr. Dent..

[6] Ferrazzano G.F., Salerno C., Bravaccio C., Ingenito A., Sangianantoni G., Cantile T. (2020). Autism spectrum disorders and oral health status: Review of the literature. Eur. J. Paediatr. Dent..

[7] Pisalchaiyong T., Trairatvorakul C., Jirakijja J., Yuktarnonda W. (2005). Comparison of the effectiveness of oral diazepam and midazolam for the sedation of autistic patients during dental treatment. Pediatr. Dent..

[8] Azevedo I.D., Ferreira M.A., Da Costa A.P., Bosco V.L., Moritz R.D. (2013). Efficacy and safety of midazolam for sedation in pediatric dentistry: A controlled clinical trial. J. Dent. Child..

[9] Soldani F., Manton S., Stirrups D.R., Cumming C., Foley J. (2010). A comparison of inhalation sedation agents in the management of children receiving dental treatment: A randomized, controlled, cross-over pilot trial. Int. J. Paediatr. Dent..

[10] Baethge C., Goldbeck-Wood S., Mertens S. (2019). SANRA-a scale for the quality assessment of narrative review articles. Res. Integr. Peer Rev..

[11] Braff M.H., Nealon L. (1979). Sedation of the autistic patient for dental procedures. ASDC J. Dent. Child..

[12] Capp P.L., De Faria M.E., Siqueira S.R., Cillo M.T., Prado E.G., De Siqueira J.T. (2010). Special care dentistry: Midazolam conscious sedation for patients with neurological diseases. Eur. J. Paediatr. Dent..

[13] Fukuta O., Braham R.L., Yanase H., Atsumi N., Kurosu K. (1993). The sedative effect of intranasal midazolam administration in the dental treatment of patients with mental disabilities. Part 1. The effect of a 0.2 mg/kg dose. J. Clin. Pediatr. Dent..

[14] Faulks D., Hennequin M., Albecker-Grappe S., Manière M.C., Tardieu C., Berthet A., Wolikow M., Droz D., Koscielny S., Onody P. (2007). Sedation with 50% nitrous oxide/oxygen for outpatient dental treatment in individuals with intellectual disability. Dev. Med. Child. Neurol..

[15] Yilmaz M.Z., Torun A.C., Baş B., Duran H., Köse H.I., Furuncuoʇlu H. (2014). Anesthetic management of dental procedures in patients with special needs: A retrospective analysis of 519 patients in North of Turkey. Int. J. Clin. Exp. Med..

[16] Macdonald A.G., Carmichael A.F. (1970). The use of diazepam for conservative dentistry in handicapped children. Anaesthesia.

[17] Allen W.A. (1984). Nitrous oxide dosage in relative analgesia. Br. Dent. J..

[18] Roberts R.E., McCrory O.F., Glasser J.H., Askew C. (1978). Dental care for handicapped children reexamined: II—Dimensions of dental practice. J. Public Health Dent..

[19] Galeotti A., Garret Bernardin A., D’Antò V., Ferrazzano G.F., Gentile T., Viarani V., Cassabgi G., Cantile T. (2016). Inhalation Conscious Sedation with Nitrous Oxide and Oxygen as Alternative to General Anesthesia in Precooperative, Fearful, and Disabled Pediatric Dental Patients: A Large Survey on 688 Working Sessions. Biomed. Res. Int..

[20] Lin I.H., Huang M.S., Wang P.Y., Huang T.S., Chong S.Y., Chen S.L.S., Tsai H.H. (2021). A comparative study of propofol alone and propofol combined with midazolam for dental treatments in special needs patients. Medicine.

[21] Eid H. (2002). Conscious sedation in the 21st century. J. Clin. Pediatr. Dent..

[22] Shapira J., Holan G., Guelmann M., Cahan S. (1992). Evaluation of the effect of nitrous oxide and hydroxyzine in controlling the behavior of the pediatric dental patient. Pediatr. Dent..

[23] Folayan M.O., Faponle A., Lamikanra A. (2002). Seminars on controversial issues. A review of the pharmacological approach to the management of dental anxiety in children. Int. J. Paediatr. Dent..

[24] Baldinelli L., Baldinelli L.G. (1991). An autism patient treated with different anesthesia and sedation techniques over 13 years. Anest. Stomatol..

[25] Stuker E.W., Eskander J.P., Gennuso S.A. (2018). Third time’s a charm: Oral midazolam vs intranasal dexmedetomidine for preoperative anxiolysis in an autistic pediatric patient. Paediatr. Anaesth..

[26] Tran J., Chen J.W., Trapp L., McCormack L. (2021). An Investigation of the Long and Short Term Behavioral Effects of General Anesthesia on Pediatric Dental Patients With Autism. Front. Oral Health.

[27] Logrieco M.D.M., Ciuffreda G.N., Sinjari B., Spinelli M., Rossi R., D’Addazio G., Lionetti F., Caputi S., Fasolo M. (2021). What Happens at a Dental Surgery When the Patient is a Child with Autism Spectrum Disorder? An Italian Study. J. Autism Dev. Disord..

[28] Yost Q., Nelson T., Sheller B., McKinney C.M., Tressel W., Chim A.N. (2019). Children with Autism Spectrum Disorder Are Able to Maintain Dental Skills: A Two-Year Case Review of Desensitization Treatment. Pediatr. Dent..

[29] Nelson T.M., Sheller B., Friedman C.S., Bernier R. (2015). Educational and therapeutic behavioral approaches to providing dental care for patients with Autism Spectrum Disorder. Spec. Care Dent..

[30] Mellado-Cairet P., Harte C., Séjourné E., Robel L. (2019). Behavioral training and mirroring techniques to prepare elective anesthesia in severe autistic spectrum disorder patients: An illustrative case and review. Paediatr. Anaesth..

